# Pathological and Prognostic Characterization of Craniopharyngioma Based on the Expression of TrkA, β-Catenin, Cell Cycle Markers, and BRAF V600E Mutation

**DOI:** 10.3389/fendo.2022.859381

**Published:** 2022-05-30

**Authors:** Cheng Xu, Songhan Ge, Juanxian Cheng, Huabin Gao, Fenfen Zhang, Anjia Han

**Affiliations:** Department of Pathology, The First Affiliated Hospital, Sun Yat-sen University, Guangzhou, China

**Keywords:** Craniopharyngioma, TrkA, β-catenin, cyclin D1, BRAF gene mutation

## Abstract

We collected 61 craniopharyngioma (CP) specimens to investigate the expression of TrkA, β-catenin, BRAF gene mutation, and NTRK1 fusion in CP. There were 37 male and 24 female individuals with a median age of 34 years (range, 4–75 years). Histologically, there were 46 cases of adamantinomatous craniopharyngioma (ACP), 14 cases of papillary craniopharyngioma (PCP), and 1 case with a mixed adamantinomatous and papillary pattern. By immunohistochemistry, we found that moderate/high TrkA expression was detected in 47% (28/60) CP and was significantly higher in adult patients (p = 0.018). Interestingly, TrkA is more expressed in “whorled epithelium” cells in ACP, similar to the localization of abnormal β-catenin. The abnormal expression rate of β-catenin was 70% (43/61), and the medium/high cyclin D1 expression rate was 73% (44/60), both of which were significantly higher in ACP than in PCP. Of the CP, 41% (21/51) had a moderate/strong P16-positive signal; 58% (34/59) showed a high Ki-67 expression, and there was a significant correlation between high Ki-67 L.I. and high tumor recurrence (p = 0.021). NTRK1 fusion was not found in CP by fluorescence *in situ* hybridization (FISH). By PCR, 26% (15/58) CP showed BRAF V600E gene mutation, which mainly occurred in PCP (100%, 14/14) except one case of mixed CP. Moreover, TrkA expression was negatively correlated with Ki-67 index and positively correlated with P16 expression. There was a significantly negative correlation between BRAF V600E mutation and abnormal β-catenin expression. Our results demonstrate for the first time that TrkA expression might occur in CP, especially in adult CP patients, and suggest that cyclin D1 could be used for ACP histological classification in addition to β-catenin and BRAF V600E mutation, while Ki-67 could be used as a marker to predict CP recurrence.

## Introduction

Craniopharyngioma (CP) is a rare WHO grade I benign neoplasm, accounting for 1.2%–4.6% of all intracranial tumors. It may originate at different positions along the sella-third ventricle axis, either from residual squamous epithelial cells of Rathke’s capsule or as a result of squamous metaplasia of the pituitary nodule (1), with two histological types: adamantinomatous craniopharyngioma (ACP) and papillary craniopharyngioma (PCP) (2, 3). Clinically, the aggressive behavior of craniopharyngioma is reflected in its tendency to infiltrate surrounding sensitive brain structures such as hypothalamus, pituitary gland, and optic chiasm. Surgical resection is the first-line treatment for craniopharyngioma, but the anatomical location of the tumor makes resection challenging. Contemporary craniopharyngioma treatment (with less aggressive surgery and/or radiation and medical therapy) generally has better outcomes and leads to long-term survival for many patients; however, tumor recurrence cannot be precluded and still occurs in approximately 50% of patients (4).

The neurotrophic tyrosine kinase receptor 1 gene (NTRK1) encodes the tropomyosin receptor kinase A (TrkA), which is a member of the tropomyosin receptor kinase (Trk) family of receptor tyrosine kinases (RTKs) (5). The Trk family plays a crucial part in the growth and function of neuronal synapses, memory development, and neuronal protection following ischemia or other types of injury (6). The binding of TrkA by nerve growth factor (NGF) causes activation of the RAS/MAPK pathway, leading to increased cellular proliferation and growth *via* ERK signaling (7). The oncogenic role of Trk was first identified in colorectal cancer (CRC) in 1986 due to an NTRK1 rearrangement (8), resulting in fusion with other genes, upregulating downstream signaling pathways independent of ligand, and promoting tumor cell proliferation and metastasis. In recent years, NTRK1 rearrangements have been confirmed in multiple cancers by next-generation sequencing, including lung cancer, soft tissue sarcoma, glioma, and malignant melanoma (9–13). Notably, NTRK1 amplification and subsequent TrkA overexpression is more common than NTRK1 rearrangement and has been reported in a variety of cancers including breast, neuroblastoma, lung cancers, pancreas, ovary, and others (14–18). In breast cancer models, ectopic overexpression of TRKA promoted tumor cell proliferation, migration, and invasion (17), while TrkA overexpression is strongly predictive of favorable outcomes in neuroblastoma (18). In addition, based on the Food and Drug Administration (FDA) approval of Larotrectinib (Vitkravi^®^) in 2018 as the first drug targeting NTRK1 rearrangement to be marketed and shown to have high efficacy and low toxicity in cancer treatment (14), we hypothesized that TrkA overexpression might provide an alternative target for cancer therapy with these Trk inhibitors. As a highly recurrent benign intracranial tumor, the Trk family protein such as TrkA has not been studied, and NTRK1 fusion has not been reported in CP.

The BRAF V600E mutation is detected in PCP (19), and CTNNB1 (β-catenin) mutations are detected in ACP (20). Cyclin D1, which is one of the downstream proteins of WNT/β-catenin signaling pathway, encoded by the CCND1 gene located on chromosome band 11q13, promotes cell cycle progression during the G1-S phase (21). There is increasing evidence that cyclin D1 is often dysregulated and serves as a biomarker of tumor phenotype and disease progression (22). P16 (also known as CDKN2A) is a tumor suppressor gene and a member of the INK family of cyclin-dependent kinase (CDK) inhibitors, which can directly regulate the activity of cyclin D1. P16 participates in the regulation of tumor cell cycle by blocking the G1 phase of the cell cycle. In CRC, increased expression of P16 may result from mutation of BRAF leading to oncogene-activated cell senescence (23). However, expressions of TRKA, cyclin D1, and P16 and their relationship with BRAF V600E mutation in craniopharyngioma have not been reported. Our study aimed to detect TrkA, β-catenin, cyclin D1, P16, and Ki-67 expression and BRAF V600E mutation and analyze their relationship with the clinicopathological features of craniopharyngioma, such as gender, age, tumor histological type, location, size, shape, consistency, calcification, adhesion strength, and degree of surgical resection.

## Materials and Methods

### Clinical Samples and Patient Information

This study was approved by the institutional review board of the authors’ institution and included 61 cases of paraffin-embedded craniopharyngioma tissues from our institute from January 2016 through December 2020. None of the patients received preoperative radiotherapy and/or chemotherapy. The clinical information such as age, gender, histological type, tumor location, tumor size, shape, consistency, calcification, adhesion strength, and surgical treatment information of each enrolled patient was collected by reviews of medical records, as shown in **Supplementary Table S1** and is summarized in **Table 1**. Histopathological classification was performed according to the WHO definition. Five major CP locations were considered: sellar–suprasellar (S/SS), sellar/suprasellar pseudo-intraventricular (S/SS-pseudo 3V), sellar/suprasellar secondary intraventricular (S/SS-secondary 3V), infundibulo-tuberal, and strictly intraventricular (strictly 3V) (1). They were divided into two groups for statistical analysis: the sellar/suprasellar CP group (S/SSC) and the intraventricular CP group (IVC). In the former group, tumors mainly grew in the sellar region and developed to the posterior and suprasellar directions, including S/SS, S/SS-pseudo 3V, S/SS-secondary 3V, and infundibulo-tuberal. The latter referred to tumors mainly growing in the third ventricle and might be accompanied by lateral ventricle enlargement. The tumor size was the maximum diameter on MRI. CP shape was classified into five categories: round, elliptical, multilobulated, pear-like, and dumbbell. Tumor consistency was classified into solid, cystic, and mixed solid–cystic by CT, MRI, or surgical records. The degree of the attachment or adhesion strength was classified into four categories: loose, tight, fusion, and replacement according to the method of Prieto et al. (24). To determine the degree of surgical resection, gross total resection (GTR) was defined as 100% macroscopic tumor resection, near-total resection (NTR) meant 95%–100% resection, and subtotal resection (STR) and partial resection (PTR) were defined as 80%-95% and < 80% resection.

**Table 1:** Clinical characteristics of craniopharyngioma.

Characteristics		No. of cases	Percentage
**Gender**			
	Male	37	60.7%
	Female	24	39.3%
**Age**			
	Median (years) 34		
	Range (years) 4–75		
**Histological type**			
	ACP	46	75.4%
	PCP	14	23.0%
	Mixed-CP	1	1.6%
**Tumor location**			
	S/SS	6	9.8%
	S/SS-pseudo 3V	9	14.8%
	S/SS-secondary 3V	13	21.3%
	Infundibulo-tuberal	26	42.6%
	Strictly 3V	7	11.5%
**Tumor size**			
	≤3 cm	25	41.0%
	>3cm	36	59.0%
**Tumor shape**			
	Pear-like	6	9.8%
	Round	14	23.0%
	Elliptical	32	52.5%
	Multilobulated	7	11.5%
	Dumbbell	2	3.3%
**Type of lesion on imaging**			
	Cystic	18	29.5%
	Mixed cystic-solid	39	63.9%
	Solid	4	6.6%
**Calcification**			
	Yes	46	75.4%
	No	15	24.6%
**Adhesion strength**			
	Loose	2	3.3%
	Tight	37	60.7%
	Invasion	21	34.4%
	Fusion	1	1.6%
**Surgical details**			
Procedure type			
	Open	49 (9 endoscope assisted)	80.0% (18.4% endoscope assisted)
	Purely endoscopic	12	20.0%
Extent of resection			
	GTR	42	68.9%
	NTR	4	6.5%
	STR/PTR	15	24.6%
**Follow-up**			
	Mean age at primary CP (years) 35.0	35.0	
	Mean age at recurrent CP (years) 37.3	37.3	
	Mean recurrence time (months) 18.6	18.6	
	Recurrence	16	26.2%

S/SS, sellar-suprasellar; 3V, the third ventricle; GTR, gross total resection; NTR, near-total resection; STR/PTR, subtotal resection/partial resection.

### Follow-Up

The patients were followed up every 3–6 months within 2 years after surgery and annually after 2 years. Follow-up content included clinical manifestations, endocrine examination, and MRI examination. Recurrence was defined as the detection of a new lesion upon gross total removal or the regrowth of tumor remnant on follow-up MRI neuroimaging. If tumor recurrence or progression is found during follow-up, appropriate additional treatment is provided, including re-surgical resection or radiation therapy.

### Immunohistochemical Staining and Evaluation

Four-micrometer-thick sections were dewaxed in xylene, hydrated in graded series of alcohol, and subjected to heat-induced antigen retrieval [10 mM sodium citrate, pH 6.0 or ethylenediaminetetraacetic acid (EDTA), pH 8.0]. All the sections were blocked with 3 % H_2_O_2_ and 1 % fetal bovine serum (FBS) for 2 h at room temperature and incubated overnight at 4°C with primary antibodies including TrkA (1:100, TA806413, OriGene, Rockville, MD, USA), β-catenin (direct, MAB-0754, MXB Technology, Fuzhou, Fujian Province, China), cyclin D1 (1:100, AF-0931, Affinity Biosciences, Cincinnati, OH, USA), P16 (1:200, AF-0228, Affinity Biosciences, Cincinnati, OH, USA), and Ki-67 (direct, ZM-0167, ZSGB-BIO, Beijing, China). Then, sections were rinsed and incubated with the biotinylated secondary antibody at room temperature for 30 min and colored by DAB, and they were then counterstained with hematoxylin for 10 s and mounted. Appropriate positive and negative controls were applied. All the slides were reviewed by two pathology doctors in a blinded manner based on a double scoring system. For inconsistent cases, the slides were re-evaluated on a multiheaded microscope to achieve consensus.

The distribution characteristics of β-catenin in the cell were judged from the three aspects of cell membrane, cytoplasm, and nucleus. The presence of brown granules on the cell membrane was considered as normal expression, while their concentration in the nucleus and/or cytoplasm was considered as abnormal expression. In this study, the staining signal in the cytoplasm and/or nuclei of tumor cells was considered positive for β-catenin expression. Total immunostaining score (IS) was calculated as the proportion score (0 = 0%, 1 < 10%, 2 = 10%–50%, 3 = 51%–80%, 4 > 80%) multiplied by the intensity score (0 = no signal, 1 = weak signal, 2 = moderate, 3 = strong). The same IS was also for cyclin D1, P16, and TrkA signal. Nuclear staining of tumor cells was assessed for cyclin D1. Nuclear and cytoplasm staining was positive for P16. Cell membrane, cytoplasmic, and/or nuclear immunostaining were assessed for TrkA expression. The above three markers were then divided into two scoring groups on the basis of the IS: absent/low expression (IS = 0–2) and moderate/high expression (IS = 3–12). For the evaluation of the proliferating index Ki-67 (labeling index), “hot spot” areas were chosen, and an average of the values on five adjacent fields (at least 500 neoplastic cells) was calculated: normally highly proliferating areas were excluded, namely, basal cells. The cutoff value was 5% tumor cells with Ki-67 positive staining. With the aim of exploring the relationship between Ki-67 and CP recurrence, Ki-67 expression was detected from primary CP samples.

### Fluorescence *In Situ* Hybridization

The split FISH probes (Guangzhou LBP Medical Technology Co., Ltd., China) were used for detecting the NTRK1 gene fusions. The slides were deparaffinized, immersed in xylene and 100% ethanol, air dried, and then microwaved in citric acid. The sections were subsequently pretreated with standard saline citrate, digested with pepsin, and immersed in graded ethanol solutions. Following drying, the probes were applied on each slide, covered with a coverslip, sealed with rubber cement, co-denatured with the target DNA, and then hybridized overnight. After washing, the sections were counterstained with 4′,6-diamidino-2-phenylindole (DAPI) and mounted with anti-fade solution. A fused or closely approximated red–green signal pattern was interpreted as a normal result, whereas splitting of the probes indicated the presence of a rearrangement. Gene rearrangement was reported to be present if ≥10% of the tumor nuclei showed split signals defined as separation of signals.

### Mutation Analysis of BRAF V600E

The QIAamp formalin-fixed paraffin-embedded (FFPE) tissue sample DNA purification kit (56404, Qiagen, Venlo, Netherlands) was used for nucleic acid extraction, and the BRAF gene V600E mutation detection kit (PCR-fluorescent probe method) was used for detection.

### Statistical Analysis

The association between the scoring group of TrkA, β-catenin, cyclin D1, P16, and Ki-67 expression, BRAF V600E mutation, and clinicopathological features was determined by X^2^ test and Fisher’s exact test. The correlation between these indicators was evaluation by Spearman’s correlation test. Statistical analysis was performed by SPSS19.0 statistical software. p-Value <0.05 was considered statistically significant. Notably, the mixed CP case was included in the ACP group for statistical analysis.

## Results

### Clinicopathological Characteristics of Patients With Craniopharyngioma

**Figure 1:**
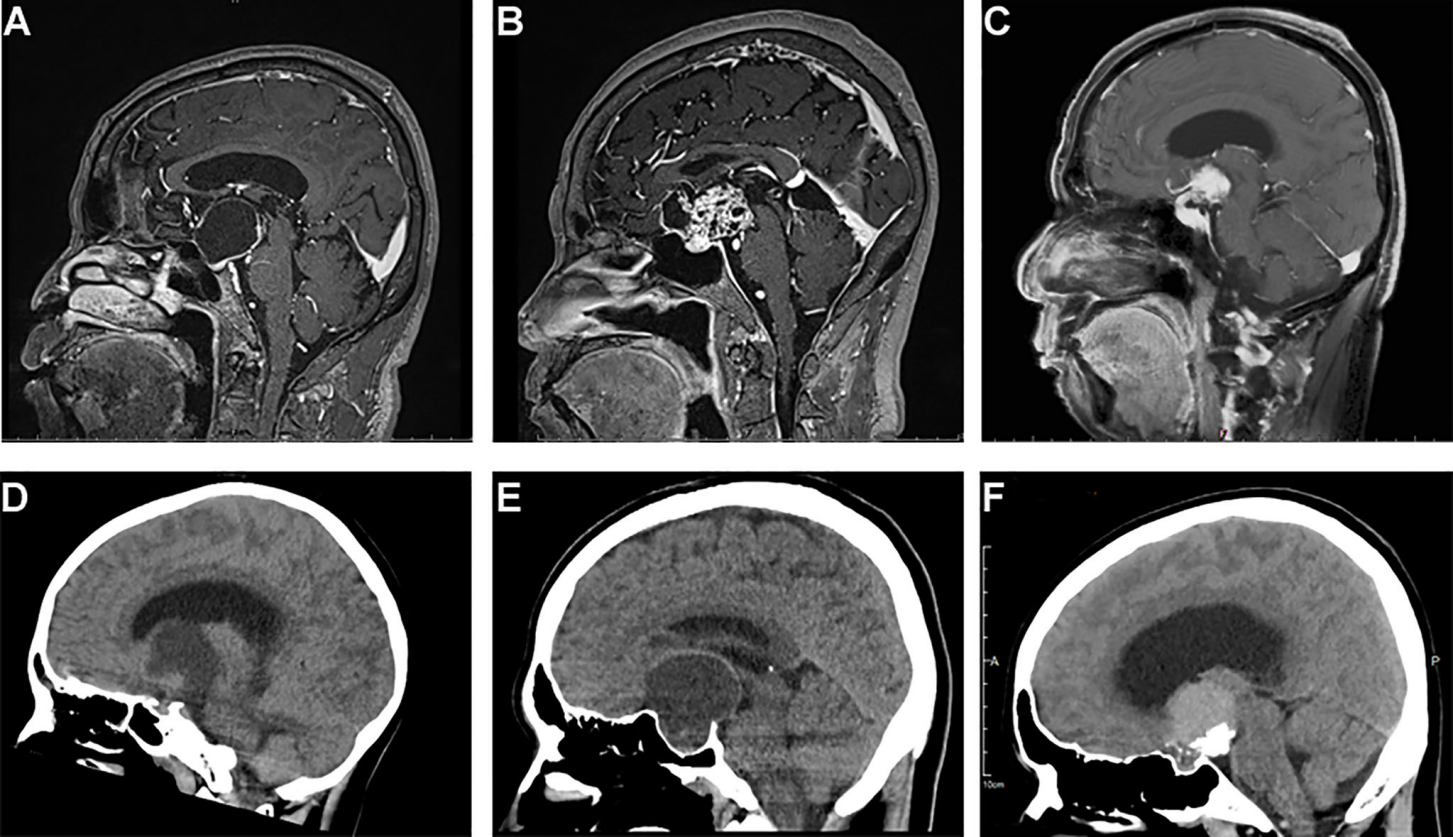
Typical sagittal MRI and CT imaging pictures of craniopharyngiomas. **(A–C)** MR images, **(A)** cystic suprasellar–pseudointraventricular PCP, **(B)** mixed solid cystic infundibulo-tuberal ACP, and **(C)** solid infundibulo-tuberal PCP. **(D–F)** CT images, **(D)** a mixed solid-cystic case of PCP, no calcification; **(E)** a mixed cystic-solid case of ACP showing peripheral eggshell calcification; and **(F)** a solid case of ACP, big block popcorn calcification.

Clinical data included patients’ gender, age, histopathology type, imaging characteristics, surgical treatment, and follow-up information (**Table 1** and **Supplementary Table S1**). A total of 61 patients were enrolled, including 37 male and 24 female patients (the ratio of male to female was 1.54:1). Patients’ age had a bimodal distribution and ranged from 4 to 75 years with the median age of 34 years. Histologically, there were 46 ACP, 14 PCP, and 1 case with a mixed adamantinomatous and papillary pattern. The most common topography of craniopharyngioma was the infundibulo-tuberal (42.6%, 26/61), followed by sellar/suprasellar secondary intraventricular (21.3%, 13/61) and sellar/suprasellar pseudo-intraventricular (14.8%, 9/61), then the strictly intraventricular (11.5%, 7/61) and sellar-suprasellar region (9.8%, 6/61). CP shape was classified into five categories: elliptical (52.5%, 2/61), round (23%, 14/61), multilobulated (11.5%, 7/61), pear-like (9.8%, 6/61), and dumbbell (3.3%, 2/61). The average tumor diameter was 3.62 cm (ranges from 1.0 to 7.6 cm). As shown in **Table 1**, 29.5% of the tumors were cystic, 6.6% were solid, and the rest (63.9%) were of mixed consistency. Tumors with a predominantly solid component were more heterogeneous, with a total of approximately 75.4% (46/61) of patients showing varying degrees of calcification. In cystic tumors, calcification of a part of the wall was present in six (33.3%, 6/18) of the patients. Of all 14 PCP patients, only 1 case had a solid tumor with popcorn-like calcification, and the rest had cystic tumor with no calcification (**Table 1**; **Figure 1**). Of the tumors, 3.3% were loose, 60.7% were tight, 34.4% were fusion, and only 1 case was a replacement. For the surgical management, as noted in **Table 1**, an open craniotomy was performed in 49 (80%) patients, of which 18.4% (9/49) underwent an endoscopy-assisted procedure; the remaining 20% underwent a purely endoscopic procedure (mainly those with cystic tumor, 7/12). GTR of the tumor was possible in 43 (70.5%) of the patients, while in 4 (6.5%), NTR was possible; only a partial or subtotal removal of the tumor was done in 14 (23%) patients. Two of these patients received conservative treatment with endocrine hormones and Ommaya reservoir prior to surgical resection (case nos. 46 and 61). The median follow-up period for this cohort was 24 months, with a range of 3–60 months. In 16 patients (26.2%), the follow-up MRI revealed tumor recurrence, while in 45 patients, there was no recurrence; the mean recurrence time was 18.6 months. One patient (case no. 41) underwent NTR tumor in April 2017 and re-surgical STR of the recurrent tumor 5 months later. In August 2018, MRI suggested a second local tumor recurrence, which was supplemented by radiotherapy, 50.4 Gy/28F.

**Figure 2:**
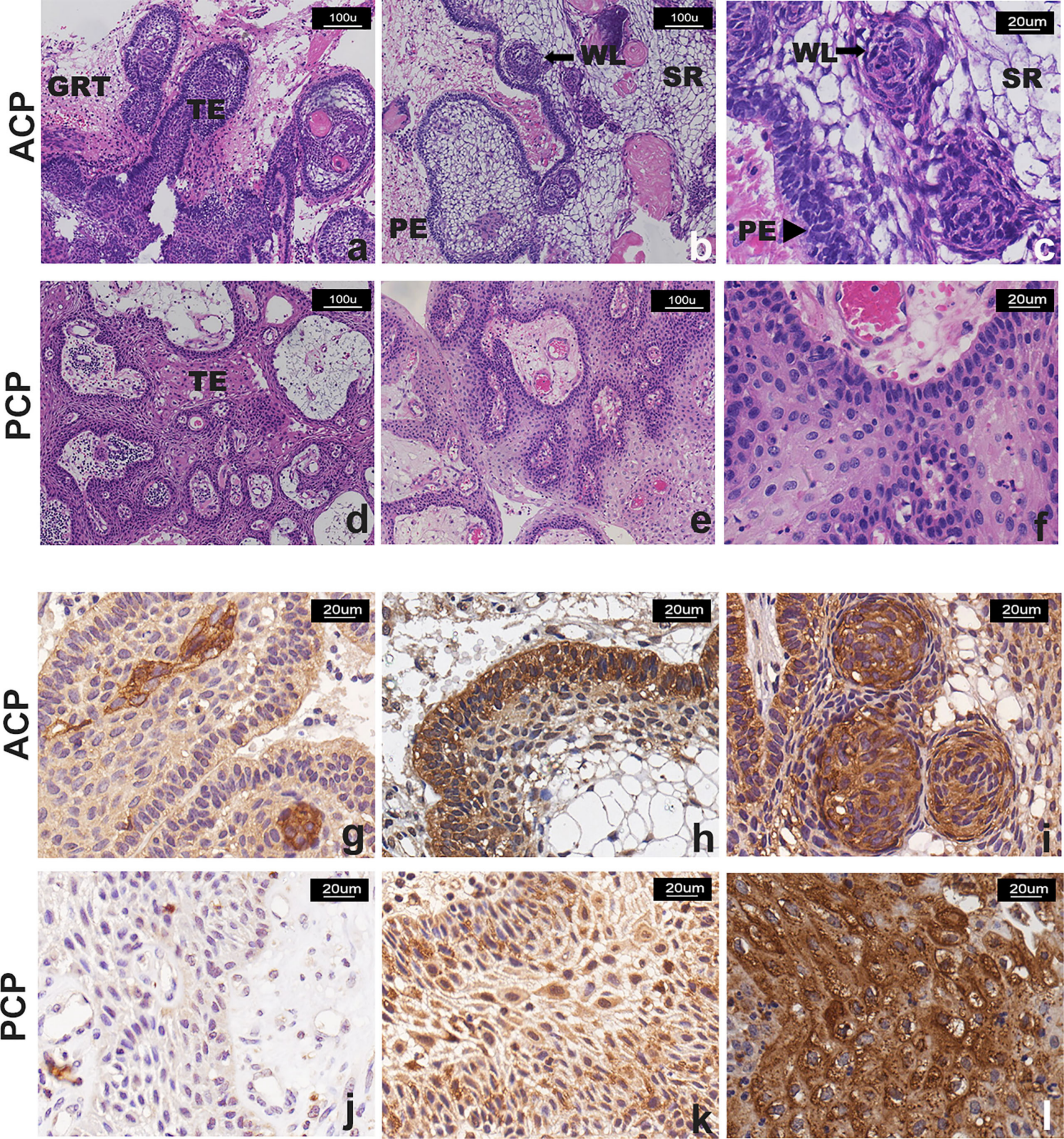
Representative images of H&E staining and TrkA immunostaining of CP patients. **(A–F)** H&E staining: **(A–C)** two cases of ACP, showing “finger-like” and “island-like” aggressive growth structures; **(D–F)** two cases of PCP, showing that the tumor was composed of non-keratinizing squamous epithelium, with visible blood vessels in the interstitial, without invasive growth structure. TE, tumor epithelium; GRT, glial reactive tissue; PE, palisading epithelium; WE, “whorled epithelium” cell groups; SR, stellate reticulum. **(G–L)** TrkA immunostaining, the signal intensity was moderate and strong and more manifested in the tumor cells that form the “whorled epithelium” structure **(G, I)** and the palisade epithelium **(H)** in ACP; the signal was observed in **(J)** <10% and **(K)** >50% with moderate and strong intensity or **(L)** strong and diffuse (51%–80% of neoplastic cells) immunoreactivity in PCP. Panels **(A, B, D–F)** were captured at 100×, and others were captured at 200× magnification.

Histologically, there were two major different types of craniopharyngioma except one case showing mixed adamantinomatous and papillary histological features. Forty-six cases of ACP were characterized by the formation of a peripheral basal cell layer of the palisading epithelium, loose aggregates of epithelial stellate cells, a “whorled epithelium” by astrocytes and cholesterol deposits. Fourteen cases of PCP were characterized by papillary and cauliflower-like morphology and composed of non-keratinizing squamous epithelium, and intercellular bridges could be seen. In addition, we found that the epithelial tumor cells in ACP had “finger-like” or “island-like” protrusions extending into the surrounding tissues with obvious glial hyperplasia, which could form strongly eosinophilic Rosenthal fibers with “whorled epithelium” cell clusters. However, all PCP cases lacked an aggressive structure (**Figures 2A–F**).

### TrkA Expression in Craniopharyngioma

Immunohistochemistry staining was used to detect the expression of TrkA protein in 60 craniopharyngioma samples except in one case where tumor tissue was too limited. Positive signals of TrkA expression were located in the tumor cell membrane, cytoplasm, and/or nucleus. Interestingly, cytoplasm and cell membrane signals were mainly found in ACP, while membranous, cytoplasm, and nuclear signals were found in PCP. Expression was mainly present in regions of the well-keratinized tumor epithelium (**Figures 2G–L**). In addition, strong signals staining was often present in the cells from the “whorled epithelium” structure. Generally, TrkA was not expressed in the stellate reticular epithelium, but expression was also observed in the palisade epithelium (**Figures 2G–I**), while positive signals of TrkA expression in PCP were diffusely distributed (**Figures 2J–L**). Thirty-two cases were categorized in the absent/low (IS = 0–2) scoring group, while 28 cases were categorized in the moderate/high group (IS > 2). Of these, 65% (18/28), 21% (6/28), and 14% (4/28) were identified as having medium/high TrkA expression in the cytoplasm, membrane, and cytoplasm, and mixed membrane–cytoplasmic–nuclear signals. First, we compared the 28 cases with high TrkA expression and the clinicopathological features of craniopharyngioma by Fisher’s exact test and found out that cytoplasmic signals of TrkA was significantly higher in the ACP or calcification group, while the mixed membrane–cytoplasmic–nuclear signal was significantly higher in the PCP or non-calcification group (**Table 2**). In addition, CP in the third ventricle was mainly a mixture of membranous, cytoplasmic, and nuclear signals with high TrkA (p = 0.000, **Table 2**). However, when only no/low and medium/high TrkA expression was analyzed, we found that TrkA expression was significantly higher in adults than in patients ≤18 years (p = 0.018, **Table 3**). No statistically significant correlation was observed between TrkA expression and age, gender, tumor histological type, location, calcification, size, and recurrence (**Table 3**).

**Table 2:** The relationship between clinical data and TrkA expression (IS≥3) in craniopharyngioma.

Variables	N = 28	TrkA IS ≥3			X²	p
		C	M+C	M+C+N		
	No. (%)	No. (%)	No. (%)	No. (%)		
**Gender**					0.342	0.871
Male	16 (57)	11 (69)	3 (19)	2 (12)		
Female	12 n(43)	7 (58)	3 (25)	2 (17)		
**Age**					1.576	0.471
≤ 18 y	3 (11)	2 (67)	0	1 (33)		
>18 y	25 (89)	16 (64)	6 (24)	3 (12)		
**Histologic type**					14.667	**0.001**
ACP	21 (75)	15 (71)	6 (29)	0		
PCP	7 (25)	3 (43)	0	4 (57)		
**Tumor location**					8.333	**0.010**
S/SSC	23 (89)	16 (70)	6 (26)	1 (4)		
IVC	5 (11)	2 (40)	0 ( 0)	3 (60)		
**Calcification**					15.852	**<0.001**
Present	21 (75)	17 (81)	4 (19)	0 (0)		
No	7 (25)	1 (14)	2 (29)	4 (57)		
**Tumor size**					4.286	0.114
≤3 cm	10 (36)	4 (40)	4 (40)	2 (20)		
>3cm	18 (64)	14 (78)	2 (11)	2 (11)		
**Recurrence**					2.098	0.417
Yes	6 (21)	5 (83)	0	1 (17)		
No	22 (79)	13 (59)	6 (27)	3 (14)		

The statistically significant finding was highlighted with bold. IVC, intraventricular craniopharyngioma; S/SSC, sellar/suprasellar craniopharyngioma; IS, immunostaining score; C, cytoplasm; M, membranous; N, nuclear.

**Table 3:** The relationship between immunohistochemical scores, BRAF V600E mutation, and clinical features of craniopharyngioma.

**Variables**	**N = 60**	**TrkA IS**		**X²**	**p**	**N = 61**	**β-catenin IS**		**X²**	**p**
		**0-2**	**≥3**				**0-2**	**≥3**		
	**No. (%)**	**No. (%)**	**No. (%)**			**No. (%)**	**No. (%)**	**No. (%)**		
**Gender**				0.179	0.793				2.284	0.184
Male	36 (60)	20 (58)	16 (42)			37 (61)	18 (49)	19 (51)		
Female	24 (40)	12 (50)	12 (50)			24 (39)	7 (29)	17 (71)		
**Age**				6.832	**0.018**				0.315	0.772
≤ 18 y	16 (27)	13 (81)	3 (19)			17 (28)	6 (35)	11 (65)		
>18 y	44 (73)	19 (45)	25 (55)			44 (72)	19 (43)	25 (57)		
**Histologic type**				0.082	1.000				26.165	**<0.001**
ACP	46 (77)	25 (57)	21 (43)			47 (77)	11 (23)	36 (77)		
PCP	14 (23)	7 (50)	7 (50)			14 (23)	14 (100)	0 (0.0)		
**Tumor location**				1.952	0.235				0.003	1.000
S/SSC	53 (88)	30 (57)	23 (43)			54 (89)	23 (43)	31 (57)		
IVC	7 (12)	2 (29)	5 (71)			7 (11)	2 (29)	5 (71)		
**Calcification**				0.000	1.000				22.538	**<0.001**
Present	45 (75)	24 (53)	21 (47)			46 (75)	11 (24)	35 (76)		
No	15 (25)	8 (52)	7 (48)			15 (25)	14 (93)	1 (7)		
**Tumor size**				0.765	0.439				0.017	1.000
≤3 cm	25 (42)	15 (60)	10 (40)			25 (41)	10 (40)	15 (60)		
>3cm	35 (58)	17 (49)	18 (51)			36 (59)	15 (42)	21 (58)		
**Recurrence**				0.737	0.162				2.09	0.084
Yes	16 (27)	10 (63)	6 (37)			16 (26)	9 (56)	7 (44)		
No	44 (73)	22 (50)	22 (50)			45 (74)	16 (36)	29 (64)		
**Variables**	**N = 60**	**Cyclin D1 IS**		**X²**	**p**	**N = 51**	**P16 IS**		**X²**	**p**
		**0-2**	**≥3**				**0-2**	**≥3**		
	**No. (%)**	**No. (%)**	**No. (%)**			**No. (%)**	**No. (%)**	**No. (%)**		
**Gender**				2.045	0.234				0.518	0.566
Male	36 (60)	12 (33)	24 (67)			31 (61)	17 (55)	14 (45)		
Female	24 (40)	4 (17)	20 (83)			20 (39)	13 (65)	7 (35)		
**Age**				0.091	0.756				0.178	0.75
≤ 18 y	17 (28)	5 (29)	12 (71)			13 (25)	7 (54)	6 (46)		
>18 y	43 (72)	11 (26)	32 (74)			38 (75)	23 (61)	15 (39)		
**Histologic type**				5.084	**0.038**				0.053	1.000
ACP	46 (77)	9 (20)	37 (80)			38 (75)	22 (58)	16 (42)		
PCP	14 (23)	7 (50)	7 (50)			13 (25)	8 (62)	5 (38)		
**Tumor location**				0.015	1				0.469	0.634
S/SSC	53 (88)	14 (26)	39 (74)			47 (78)	27 (57)	20 (43)		
IVC	7 (12)	2 (29)	5 (71)			4 (22)	3 (75)	1 (25)		
**Calcification**				4.091	0.088				0.012	1.000
Present	45 (75)	9 (20)	36 (80)			36 (71)	21 (58)	15 (42)		
No	15 (25)	7 (47)	8 (53)			15 (29)	9 (60)	6 (40)		
**Tumor size**				0.057	1.000				1.996	0.253
≤3 cm	24 (40)	6 (25)	18 (75)			23 (45)	16 (70)	7 (30)		
>3cm	36 (60)	10 (28)	26 (72)			28 (55)	14 (50)	14 (50)		
**Recurrence**				0.455	0.204				0.178	0.75
Yes	15 (25)	5 (33)	10 (67)			13 (25)	7 (54)	6 (46)		
No	45 (75)	11 (25)	34 (75)			38 (75)	23 (61)	15 (39)		
**Variables**	**N = 59**	**Ki-67 L.I.**		**X²**	**p**	**N = 58**	**BRAF V600E mut**		**X²**	**p**
		**0-4**	**≥5**				**Negative**	**Positive**		
	**No. (%)**	**No. (%)**	**No. (%)**			**No. (%)**	**No. (%)**	**No. (%)**		
**Gender**				0.459	0.592				1.806	0.230
Male	36 (61)	14 (39)	22 (61)			34 (57)	23 (68)	11 (32)		
Female	23 (39)	11 (48)	12 (52)			24 (43)	20 (83)	4 (17)		
**Age**				1.643	0.252				1.656	0.308
≤ 18 y	17 (29)	5 (29)	12 (71)			15 (26)	13 (87)	2 (13)		
>18 y	42 (71)	20 (48)	22 (51)			43 (74)	30 (70)	13 (30)		
**Histologic type**				0.437	0.549				52.903	**<0.001**
ACP	45 (76)	18 (40)	27 (60)			44 (76)	43 (98)	1 (2)		
PCP	14 (24)	7 (50)	7 (50)			14 (24)	0 (0)	14 (100)		
**Tumor location**				0.159	0.691				1.199	0.360
S/SSC	53 (90)	22 (42)	31 (58)			51 (88)	39 (76)	12 (24)		
IVC	6 (10)	3 (50)	3 (50)			7 (12)	4 (57)	3 (43)		
**Calcification**				2.559	0.137				30.928	**<0.001**
Present	44 (75)	16 (26)	28 (64)			43 (74)	40 (93)	3 (7)		
No	15 (25)	9 (67)	6 (33)			15 (26)	3 (20)	12 (80)		
**Tumor size**				1.345	0.291				0.863	0.381
≤3 cm	24 (41)	8 (29)	16 (71)			25 (43)	17 (68)	8 (32)		
>3cm	35 (59)	17 (49)	18 (51)			33 (57)	26 (79)	7 (21)		
**Recurrence**				4.374	**0.021**				2.109	0.095
Yes	16 (27)	3 (19)	13 (81)			15 (26)	9 (60)	6 (40)		
No	43 (73)	21 (49)	22 (51)			43 (74)	34 (79)	9 (21)		

The statistically significant finding was highlighted with bold. IVC, intraventricular craniopharyngioma; S/SSC, sellar/suprasellar craniopharyngioma; IS, immunostaining score; L.I., labeling index.

### β-Catenin, Cyclin D1, P16, and Ki-67 Expression in Craniopharyngioma

**Figure 3:**
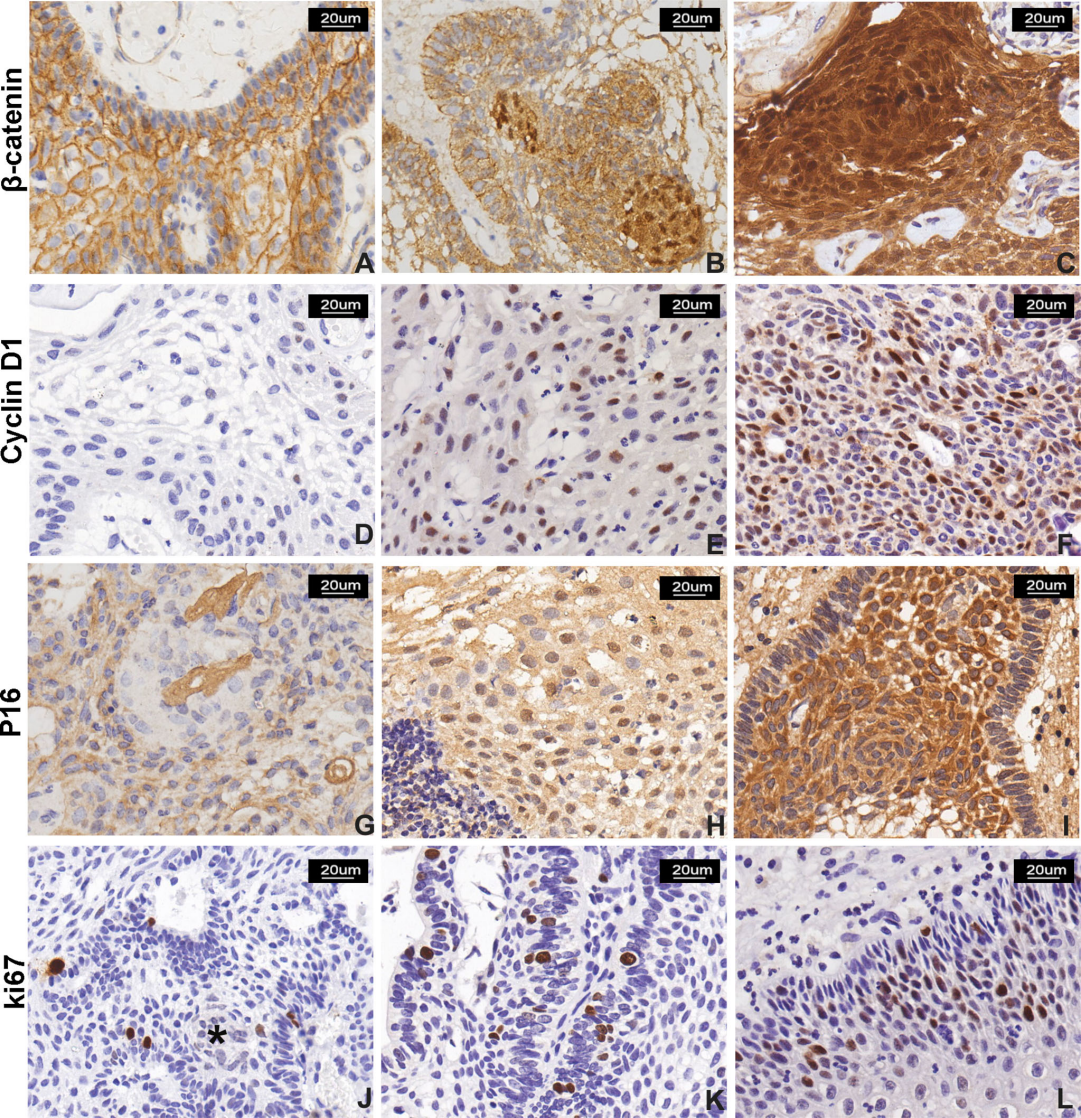
β-Catenin, cyclin D1, P16, and Ki-67 expression in CP. **(A–C)** β-Catenin immunostaining score: **(A)** normal membranous staining in stratified squamous epithelial cells in PCP, **(B)** moderate and strong nuclear signal in 10%–50% and **(C)** >50% of neoplastic cells in two ACP cases, respectively. The signal was more represented in cells forming “whorled epithelium” structures. **(D–F)** Cyclin D1 immunostaining score: low nuclear signal observed in <10% of neoplastic cells (**D**, a case of PCP); moderate signal observed in 10%–50% of cells (**E**, a case of PCP); and strong reactivity in >50% of neoplastic cells (**F**, a case of ACP). **(G–I)** P16 immunostaining score: **(G)** signal with low intensity observed in <10% of neoplastic cells in PCP and **(H)** moderate intensity observed in 10%–50% of neoplastic cells in PCP; and **(I)** moderate and strong and diffuse (51%–80% of neoplastic cells) immunoreactivity in ACP. **(J–L)** Ki-67 labeling index: low proliferative index Ki-67 (<5%), and “whorled epithelium” structure (*) **(J)** extremely low Ki-67 index, **(K)** high Ki-67 index, and **(I)** Ki-67 index calculated as 15% in a case of PCP. All pictures were captured at 200× magnification.

We further analyzed β-catenin expression in all 61 cases of craniopharyngioma. The positive and abnormal expressions of β-catenin in CP was 100% (61/61) and 70% (43/61), respectively. In ACP, β-catenin expression was found in the membranes of palisading tumor cells; some tumor cells in the cytoplasm and nucleus were abnormal β-catenin positive. The distribution of cells with abnormal β-catenin expression was mainly located in tumor cells forming the “whorled epithelium” structure, which was consistent with that of TrkA strongly positive expression. In all 14 PCP cases, β-catenin positive staining was evenly expressed in the membrane of most tumor parenchymal cells, and no β-catenin positive signals were found in the cytoplasm and/or nucleus of tumor cells (**Figures 3A–C**). The probability of abnormal expression of β-catenin with moderate/high signal in ACP and PCP was 77% (36/47) and 0% (0/14), respectively. Statistical analysis revealed that the abnormal expression of β-catenin was significantly correlated with the histological type and tumor calcification of craniopharyngioma; there were more β-catenin abnormal expression in ACP and calcification groups (p <0.001, **Table 3**), but there was no significant correlation with other clinicopathological variables.

Abnormal cell cycle regulation is an important event of tumorigenesis; we evaluate the cell-cycle-associated markers including cyclin D1, P16, and Ki-67 expression in craniopharyngioma by immunohistochemistry staining. Cyclin D1 was evaluated in 60 cases. The signal was positively expressed in the nucleus. In ACP, positive cells are widely distributed in each layer including palisade epithelial cells, stellate cells, and “whirl-shaped” cell clusters; in PCP, cyclin D1-positive cells are also evenly distributed in the epithelial cells of the tumor (**Figures 3D–F**). The positive expression rate of cyclin D1 in ACP and PCP was 100% (46/46) and 93% (13/14), respectively. Sixteen cases were assigned to the absent/low (IS = 0–2) scoring group, while 44 cases were assigned to the moderate/high scoring group (IS > 2). The medium/high expression rate of cyclin D1 in CP was 73% (44/60). There was a higher cyclin D1 expression trend in ACP than that in PCP. Cyclin D1 expression was significantly related to the histological type of craniopharyngioma (p = 0.038, **Table 3**).

P16 displayed nuclear and cytoplasmic immunostaining with variable distribution in both ACP and PCP (**Figures 3G–I**). The total medium/high positive expression rate of P16 in CP was about 41% (21/51). No significant association between P16 expression and clinicopathological variables was observed.

Ki-67 is a nuclear protein and a marker for proliferation index. Ki-67 immunostaining was performed in 59 primary cases of craniopharyngioma with a mean value of 6% (median, 5%; range, 1%–30%); about 58% (34/59) of patients showed a high expression of Ki-67. We found that Ki-67-positive cells were mainly located in the peripheral basal cell layer of the palisade epithelium rather than that of the “whorled epithelium” structure or ghost cell in ACP, while Ki-67 expression was diffused in PCP (**Figures 3J–L**). The Ki-67 L.I. in ACP and PCP was 6.0 ± 0.72% and 6.7 ± 1.23%, respectively, and there was no significant difference in Ki-67 marker index between the two groups. However, there was a significant difference between Ki-67 L.I. with tumor recurrence (p = 0.021, **Table 3**). The result suggests that Ki-67 could be a marker for predicting the biological behavior of craniopharyngioma. Next, we further analyzed Ki-67 L.I. in recurrent ACP and PCP, which were 7.8 ± 2.36% and 10.4 ± 2.11%, respectively, suggesting that Ki-67 had no significant difference in CP recurrence between different histological types (p = 0.453, **Supplementary Table S2**). The degrees of tumor adhesion and tumor removal are well-known therapeutic and pathological factors, so we further analyzed the correlation between Ki-67 and CP recurrence with these two factors and found that Ki-67 L.I. in the GTR group was significantly higher than in the remaining incomplete groups (p = 0.027, **Supplementary Table S3**), and the degree of adhesion strength and tumor removal were both closely related to CP recurrence (p <0.001, **Supplementary Table S3**).

We compared the correlation between the expression of these proteins in CP, and the results showed that TrkA expression was negatively correlated with Ki-67 index and positively correlated with P16 expression in CP. Abnormal β-catenin expression was positively correlated with cyclin D1 expression and was negatively correlated with Ki-67 index. In addition, Ki-67 index was also negatively correlated with P16 expression in CP (**Table 4**).

**Table 4:** Correlation of TrkA, β-catenin, cell cycle markers immunohistochemical score, and BRAF V600E mutation in craniopharyngioma.

		TrkA	β-catenin	Cyclin D1	P16	Ki-67	BRAF V600E
TrkA	r	1.000	0.088	−0.070	0.338	−0.441	0.023
	P	–	0.503	0.598	**0.016**	**<0.001**	0.867
β-catenin	r	0.088	1.000	0.497	0.000	−0.272	−0.666
	P	0.503	–	**<0.001**	0.999	**0.034**	**<0.001**
Cyclin D1	r	−0.070	0.497	1.000	-0.148	0.2	−0.222
	P	0.598	**<0.001**	–	0.310	0.126	0.096
P16	r	0.338	0.000	−0.148	1.000	−0.463	0.053
	P	**0.016**	0.999	0.310	–	**0.001**	0.732
Ki-67	r	−0.441	−0.272	0.2	−0.463	1.000	0.142
	P	**<0.001**	**0.034**	0.126	**0.001**	–	0.286
BRAF V600E	r	0.023	−0.666	−0.222	0.053	0.142	1.000
	P	0.867	**<0.001**	0.096	0.732	0.286	–

The statistically significant finding was highlighted with bold.

### NTRK1 Gene Fusions in Craniopharyngioma

**Figure 4:**
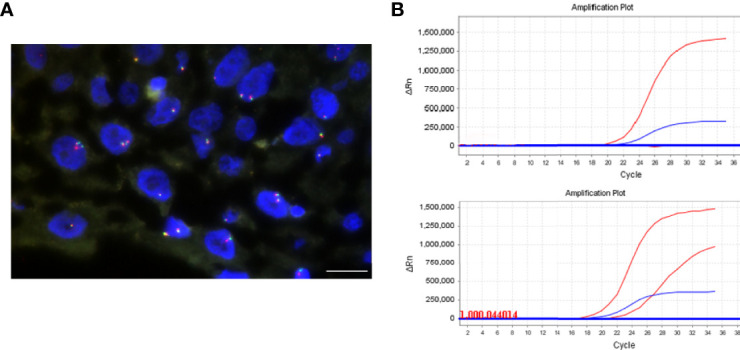
NTRK1 rearrangements and BRAF V600E mutation detection. **(A)** FISH assay using split FISH probes for confirming NTRK1 rearrangements. No split red and green signals were observed. **(B)** BRAF V600E mutation was detected by real-time PCR. The top is negative, and bottom is positive. The scale bar = 20 μm.

We further detected NTRK1 gene fusions in CP with positive TrkA expression by FISH (**Figure 4A**). In these 28 cases, signal was not identified in 12 cases because of tissue exhaustion, and the remaining 16 cases were negative for NTRK1 gene fusions.

### BRAF V600E Mutation in Craniopharyngioma

The BRAF mutation was detected by PCR (**Figure 4B**). The total mutation rate of the BRAF V600E gene in this study was 26% (15/58). All ACP patients were negative for the BRAF V600E mutation (100%, 43/43), while PCP patients were positive (100%, 14/14), and the mixed CP was diffusely positive for BRAF V600E mutation. There was a significant difference in BRAF V600E mutation between ACP and PCP (p <0.001, **Table 3**). In addition, there were more BRAF V600E mutation in no-calcification CP (p < 0.001, **Table 3**). Moreover, there was a significant negative correlation between BRAF V600E mutation and abnormal β-catenin expression in CP (**Table 4**).

## Discussion

Difficulty in surgical removal and high recurrence rate are the distinguishing characteristics of craniopharyngioma from other benign pituitary tumors. It is important to identify the new molecular biomarkers that influence biological and clinical behaviors of craniopharyngioma.

There are two histological variants of craniopharyngiomas: adamantinomatous type (ACP) and papillary type (PCP). Several clinical studies have shown that the postoperative recurrence rate and prognosis of ACP are worse than those of the PCP (25), and the invasive growth characteristics of the former may lead to adverse effects. Studies have found that the mutation frequency of CTNNB1 gene in ACP is 75%–96% (20), the BRAF V600E mutation in PCP is 95% (19), and the CTNNB1 and BRAF V600E mutations are mutually exclusive. Another study showed that BRAF V600E and CTNNB1 mutations may coexist in ACP tumors (26). As in our study, β-catenin was highly expressed in ACP, especially the presence of the β-catenin nuclear signal of tumor cells that form a “whorled epithelium” structure, while PCP showed a 100% mutation rate of BRAF V600E. It is worth noting that a 7-year-old female patient (case no. 43), who had both diffusely positive β-catenin and BRAF V600E gene mutations. This occasional case showed mixed adamantinomatous and papillary histological features, which might be more appropriately called mixed histological CP (4). As systematic studies of hundreds of cases in the 1970s and 1990s showed, approximately 33% of CP exhibited mixed histological features (27).

With the development of multi-omics data analysis tools and sequencing methods (28, 29), NTRK1 gene fusions have been found in colorectal cancer, glioma, and lung cancer (7, 9, 30). Under normal circumstances, NTRK1 encodes the TrkA receptor, which is a member of the Trk family of RTKs, and plays an important role in embryonic development and the maintenance of normal nervous system functions. Its activation induces the Ras/MAPK, PI3K/AKT, and PLC-gamma signaling pathways (7, 31). When NTRK gene is fused with other genes, the activity of Trk kinase is deregulated, which promotes the proliferation and metastasis of tumor cells. Moreover, TrkA overexpression has been found in a variety of cancers (17, 18). For example, in breast cancer models, ectopic overexpression of TrkA is associated with proliferation, migration, and invasion of tumor cells (17), and TrkA overexpression is closely associated with a favorable prognosis of neuroblastoma (18). Notably, the specific staining pattern of Trk expression is related to the subcellular localization of the fusion partner (32). For example, in the case of the NTRK1-LMNA fusion, immunostaining was observed in the nuclear membrane; however, in the case of the NTRK1-TPM fusion, mixed cytoplasmic and membranous staining was observed (32). In this study, we simultaneously performed IHC and FISH, first using anti-TrkA monoclonal antibody to detect the expression of TrkA in CP and further using split FISH probe to detect NTRK1 gene fusion in CP with positive TrkA expression. Specifically, approximately 47% (28/60) of craniopharyngioma showed medium/high expression of TrkA staining. In addition, all TrkA-positive CP samples displayed mixed cytoplasmic, nuclear, and membranous staining patterns. Unfortunately, FISH results have not found NTRK1 gene fusion. Therefore, given the high frequency of staining observed in this study, we speculate that its expression pattern seems most likely to correspond to the epigenetically regulated expression of TrkA in CP rather than to oncogenic rearrangements. Despite the limitations of our present study (small sample size from a single institution), we identified a stronger TrkA expression in adult CP patients than that in non-adult patients. Notably, our results demonstrate for the first time that TrkA expression has been found to be a substantial proportion of CP and is more common in ACP, and the expression may still be biologically interesting for tumor pathogenesis.

β-Catenin is a key player in canonical WNT/β-catenin signaling and has a dual function. When expressed in the cell membrane, it assists E-cadherin to perform a homogeneous adhesion function to inhibit tumor metastasis; when expressed in the cytoplasm and nucleus, it functions as a downstream element of the WNT/β-catenin pathway and participates in certain target genes (such as cyclin D1) that promote cell proliferation. Both of these effects are related to the occurrence and development of tumors. WNT signaling is one of the most important pathways involved in stem cell maintenance (33). There is a striking amount of evidence pointing that tumor cells with nuclear β-catenin expression have the characteristics of tumor stem cells (34), with the ability of self-renewal and multidirectional differentiation (35). In this study, abnormal expression of β-catenin was found in 70% of ACP, manifested as accumulation in the nucleus and cytoplasm, and the abnormally expressed cells were mainly distributed in the whorled epithelium but rarely in peripheral palisade-like structures, while normal membrane expression of β-catenin was found in all PCP cases. It suggests that β-catenin expression could be used as a molecular basis for differentiating two pathological types of CP, and the whorled epithelium may be related to the higher invasive biology of ACP (36, 37).

The immunohistochemical study of TrkA and β-catenin in this study showed that the distribution of these two markers in tumor tissue is similar, that is, the cells with abnormal expression of β-catenin in the “whorled epithelium” showed strong positive TrkA expression, and the palisade epithelium cells with normal membrane expression of β-catenin showed weak to moderate positive expression of TrkA. The localization of TrkA in these regions is interesting and may provide additional evidence for the unique differentiation state of these cells as reported (38). In addition, studies have shown that TrkA can directly phosphorylate β-catenin at Tyr142, which is known to promote β-catenin translocation from the membrane to the cytoplasm (39). The above results suggest that TrkA may interact with WNT/β-catenin signaling in regulating the invasion of CP.

In addition, studies have shown that the overexpression of cyclin D1 is related to the occurrence and development of tumors, shortening the survival period of patients and increasing the recurrence rate (40). Cyclin D1 overexpression has been reported in lung cancer, esophageal squamous cell carcinoma, pancreatic cancer, breast cancer, and head and neck tumors (41–44). The positive rate of cyclin D1 expression in CP was 98% (59/60), and its expression level was significantly higher in ACP than PCP, suggesting that this protein might be involved in the invasion process of ACP as a downstream gene of β-catenin.

P16 is encoded by the tumor suppressor gene CDKN2A (45), which is the first reported tumor suppressor gene family member, and can be detected by IHC. P16 protein is an important component of cell cycle checkpoints, which prevents cell proliferation by preventing the progression from G1 to S phase (46). The medium/high positive expression rate of P16 in ACP and PCP was 42% (16/38) and 38% (5/13), respectively. P16 positivity had no statistically significant association between clinicopathological variables (age, gender, histological type, tumor location, size, and recurrence). These findings may be due to the heterogeneous distribution of P16 positivity within each sample, which makes the results heavily user dependent and unreliable.

Ki-67 is a reliable marker widely used to measure proliferative activity (47). At present, many studies have used immunohistochemical methods to analyze the expression of the proliferation index Ki-67 in craniopharyngiomas, but they have not yet reached a unified standard (48, 49). A possible explanation for discrepancies between these studies is that the proliferative activity of CP may fluctuate dramatically over time. In our study, Ki-67 levels were measured in 59 primary craniopharyngiomas, and a statistically significant association between high Ki-67 (≥5%) and tumor recurrence was found, consistent with the study performed by Guadagno et al. (49). However, Ki-67 was not significantly associated with the histological type of CP recurrence, which may be related to a smaller sample size. In addition, we also found that Ki-67 positivity was most prominent and uniform in the peripheral/basal “palisading” epithelium, but not in whorled cells, as previously described in isolated case reports that Ki-67 was associated with rapid tumor recurrence (50). In our cohort study, it was found that CP recurrence was closely related to the degree of adhesion strength (p < 0.001), and about 81% (13/16) of recurrence patients have fusion or replacement features; presumably, the division and proliferation of these palisade-like cells increased tumor invasion and infiltration. Furthermore, we also found that the recurrence of CP was closely related to the degree of tumor resection (p < 0.001). Among the recurrent CP patients, about 87.5% (14/16) underwent subtotal/partial resection, and residual tumor cells that were incompletely resected due to excessive tumor adhesion and invasion of surrounding brain tissue may also lead to tumor recurrence.

## Conclusions

Our results demonstrate for the first time that TrkA expression may occur in CP, especially in the ACP variant, and that TrkA expression tends to be significantly higher in adult CP patients. Particularly, ACP or calcified CP had higher cytoplasmic TrkA signals, while CP located in the third ventricle had high expression of mixed membrane, cytoplasmic, and nuclear signals. Second, the expression of cyclin D1 in ACP was significantly higher than that in PCP, highlighting that cyclin D1 could be used in the histological classification of CP except for β-catenin and BRAF V600E mutations. Moreover, high Ki-67 can be used as a marker to predict CP recurrence. Finally, we provide reliable evidence supporting the true existence of mixed CP with both adamantinomatous and papillary histological features.

## Data Availability Statement

The raw data supporting the conclusions of this article will be made available by the authors, without undue reservation.

## Ethics Statement

The studies involving human participants were reviewed and approved by the Ethics Committee of Clinical Research and Laboratory Animals of the First Affiliated Hospital of Sun Yat-sen University. The patients/participants provided their written informed consent to participate in this study.

## Author Contributions

CX and AH designed the research. CX, SG, HG, JC, and FZ performed the research. CX analyzed the data. CX and AH wrote the paper. All authors contributed to the article and approved the submitted version.

## Conflict of Interest

The authors declare that the research was conducted in the absence of any commercial or financial relationships that could be construed as a potential conflict of interest.

## Publisher’s Note

All claims expressed in this article are solely those of the authors and do not necessarily represent those of their affiliated organizations, or those of the publisher, the editors and the reviewers. Any product that may be evaluated in this article, or claim that may be made by its manufacturer, is not guaranteed or endorsed by the publisher.
