# Supplementary material for: Pathological and Prognostic Characterization of Craniopharyngioma Based on the Expression of TrkA, β-Catenin, Cell Cycle Markers, and BRAF V600E Mutation
Source: Front Endocrinol (Lausanne). 2022 May 30;13:859381. doi: 10.3389/fendo.2022.859381 (PMC9190302; doi:10.3389/fendo.2022.859381)
Supplement: Supplementary file 1 [file DataSheet_1.pdf]

Pathological and prognostic characterization of Craniopharyngioma based on the expression of TrkA, β-catenin, cell cycle markers and BRAF V600E mutation

Supporting Information

Cheng Xu#, Songhan Ge#, Juanxian Cheng, Huabing Gao, Fenfen Zhang, and Anjia Han *

[1]Department of Pathology, The First Affiliated Hospital, Sun Yat-sen University, Guangzhou 510080, Guangdong, P. R. China

# Cheng Xu and Songhan Ge contributed equally to this work.

Runing title: TrkA, β-catenin, and BRAF V600E mutation in craniopharyngioma

*Corresponding Author: Anjia Han, MD

Department of Pathology, The First Affiliated Hospital, Sun Yat-sen University, 58, Zhongshan Road II, Guangzhou 510080, China.

Tel/Fax: 8620-87332235

E-mail: hananjia@mail.sysu.edu.cn

Table S1

Table S1. The clinical characteristics of each enrolled craniopharyngioma patient.

Case No.	Histological type	Gender, Age (years)	Tumor location	Tumor size (cm)	Tumor shape	Tumor Consistency	Calcification		Adhesion strength	Surgical details		Additional treatment	Follow-up (months)	Recurrence	Recurrence time (months)
							Present	Degree		Procedure type	Extent of resection				
1	ACP	F, 26	S/SS-secondary 3V	3.0	Pear-like	Mixed	No	/	Tight	Open (endoscope assisted)	GTR		10	No	/
2	ACP	M, 57	S/SS-secondary 3V	5.0	Elliptical	Mixed	No	/	Tight	Open	GTR		48	No	/
3	ACP	M, 68	Strictly 3V	3.1	Multilobulated	Solid	Present	Punctate	Tight	Open	GTR		36	No	/
4	ACP	F, 4	S/SS-secondary 3V	4.0	Elliptical	Cystic	Present	Eggshell	Tight	Purely endoscopic	GTR		30	No	/
5	ACP	F, 7	S/SS-pseudo 3V	5.3	Elliptical	Mixed	Present	Eggshell	Invasion	Open	GTR		6	No	/
6	ACP	F, 37	Strictly 3V	3.5	Elliptical	Cystic	Present	Punctate	Tight	Purely endoscopic	STR/PTR		24	No	/
7	ACP	M, 47	Infundibulo-tuberal	2.7	Dumbbell	Mixed	Present	Eggshell	Invasion	Purely endoscopic	STR/PTR		12	No	/
8	ACP	M, 14	Infundibulo-tuberal	4.0	Multilobulated	Mixed	Present	Eggshell	Invasion	Purely endoscopic	GTR		30	No	/
9	ACP	M, 5	S/SS-pseudo 3V	7.6	Multilobulated	Mixed	Present	Big, popcorn	Tight	Open	GTR		9	No	/
10	ACP	M, 11	Infundibulo-tuberal	3.0	Elliptical	Mixed	Present	Eggshell	Tight	Open	GTR		12	No	/
11	ACP	M, 15	Infundibulo-tuberal	3.5	Elliptical	Mixed	Present	Punctate	Tight	Open (endoscope assisted)	GTR		3	No	/
12	ACP	M, 38	Infundibulo-tuberal	3.4	Pear-like	Mixed	Present	Punctate	Tight	Open	GTR		12	No	/
13	ACP	M, 21	S/SS-secondary 3V	5.0	Elliptical	Mixed	Present	Big, popcorn	Invasion	Open (endoscope assisted)	GTR		24	No	/
14	ACP	M, 18	S/SS-pseudo 3V	3.8	Elliptical	Mixed	Present	Patchy	Invasion	Open	GTR		12	No	/
15	ACP	F, 14	Infundibulo-tuberal	2.0	Round	Mixed	Present	Big, popcorn	Tight	Open	GTR		48	No	/
16	ACP	F, 51	S/SS-secondary 3V	4.0	Elliptical	Mixed	Present	Punctate	Tight	Open	GTR		18	No	/
17	ACP	F, 56	S/SS-pseudo 3V	3.4	Multilobulated	Mixed	Present	Punctate	Invasion	Open (endoscope assisted)	STR/PTR		36	Yes	6
18	ACP	M, 51	Infundibulo-tuberal	2.6	Round	Mixed	Present	Eggshell	Tight	Open	GTR		24	No	/
19	ACP	F, 55	S/SS-secondary 3V	2.4	Elliptical	Solid	Present	Eggshell	Tight	Open	GTR		30	No	/
20	ACP	F, 58	S/SS	1.0	Elliptical	Mixed	Present	Punctate	Tight	Open	GTR		24	No	/
21	ACP	F, 55	Infundibulo-tuberal	1.9	Multilobulated	Mixed	Present	Eggshell	Tight	Open	GTR		48	No	/
22	ACP	F, 60	S/SS-pseudo 3V	4.0	Multilobulated	Mixed	Present	Big, popcorn	Invasion	Open	GTR		21	No	/
23	ACP	M, 55	S/SS-secondary 3V	2.0	Round	Solid	Present	Punctate	Loose	Open	GTR		20	No	/
24	ACP	F, 48	Infundibulo-tuberal	2.5	Round	Cystic	Present	Patchy	Invasion	Open (endoscope assisted)	STR/PTR		30	No	/
25	ACP	F, 62	S/SS	2.2	Elliptical	Mixed	Present	Punctate	Tight	Open	GTR		35	No	/
26	ACP	M, 45	Strictly 3V	3.7	Pear-like	Mixed	Present	Punctate	Tight	Open	GTR		24	No	/
27	ACP	M, 23	Infundibulo-tuberal	3.8	Elliptical	Mixed	Present	Big, popcorn	Tight	Open	GTR		29	No	/
28	ACP	F, 21	Infundibulo-tuberal	2.1	Elliptical	Mixed	Present	Punctate	Tight	Open (endoscope assisted)	NTR		36	No	/
29	ACP	M, 53	Infundibulo-tuberal	3.2	Elliptical	Mixed	Present	Eggshell	Tight	Open	GTR		24	No	/
30	ACP	M, 8	S/SS-pseudo 3V	6.4	Elliptical	Mixed	Present	Eggshell	Invasion	Open	STR/PTR		36	Yes	30
31	ACP	M, 32	Infundibulo-tuberal	3.2	Elliptical	Mixed	Present	Punctate	Tight	Open	GTR		24	No	/
32	ACP	M, 24	S/SS	2.7	Elliptical	Mixed	Present	Eggshell	Tight	Purely endoscopic	GTR		28	No	/
33	ACP	F, 10	S/SS-secondary 3V	3.8	Pear-like	Mixed	No	/	Tight	Open	GTR		30	No	/
34	ACP	F, 14	Infundibulo-tuberal	3.9	Round	Cystic	Present	Patchy	Tight	Purely endoscopic	STR/PTR		30	No	/
35	ACP	F, 11	Strictly 3V	4.5	Elliptical	Mixed	Present	Punctate	Tight	Open	GTR		36	No	/
36	ACP	M, 17	Infundibulo-tuberal	4.3	Round	Mixed	Present	Big, popcorn	Tight	Open	GTR		36	No	/
37	ACP	M, 45	Infundibulo-tuberal	4.7	Elliptical	Mixed	Present	Punctate	Tight	Open	GTR		30	No	/

38	ACP	M, 12	Infundibulo-tuberal	3.7	Elliptical	Cystic	Present	Punctate	Invasion	Open	GTR		24	No	/
39	ACP	M, 14	Infundibulo-tuberal	4.3	Round	Cystic	Present	Patchy	Invasion	Purely endoscopic	NTR		17	yes	5
40	ACP	M, 24	Infundibulo-tuberal	3	Elliptical	Mixed	Present	Eggshell	Invasion	Purely endoscopic	STR/PTR		48	yes	42
41	ACP	F, 75	S/SS-secondary 3V	4.5	Round	Mixed	Present	Punctate	Tight	Open (endoscope assisted)	NTR	STR (5months after the first operation)<br>RT (7months after the secondary operation)	11	yes	5
42	ACP	M, 33	Infundibulo-tuberal	4	Elliptical	Mixed	Present	Patchy	Invasion	Open	GTR		24	yes	24
43	Mixed-CP	F, 7	Infundibulo-tuberal	2.8	Elliptical	Mixed	Present	Big, popcorn	Invasion	Open	STR/PTR		12	yes	7
44	ACP	M, 23	S/SS	2.5	Dumbbell	Mixed	Present	Patchy	Invasion	Purely endoscopic	STR/PTR		30	yes	24
45	ACP	F, 36	Infundibulo-tuberal	2.9	Elliptical	Mixed	Present	Patchy	Invasion	Open (endoscope assisted)	GTR		12	yes	6
46	ACP	M, 36	Infundibulo-tuberal	7.3	Pear-like	Mixed	Present	Punctate	Tight	Open	STR/PTR	pre OMMAY CI	60	yes	60
47	ACP	F, 35	S/SS	2.7	Elliptical	Mixed	Present	Eggshell	Invasion	Open (endoscope assisted)	STR/PTR		24	yes	6
48	PCP	M, 60	S/SS-secondary 3V	3.0	Elliptical	Mixed	Present	Patchy	Invasion	Open	STR/PTR		45	Yes	42
49	PCP	M, 34	Infundibulo-tuberal	2.6	Elliptical	Cystic	No	/	Tight	Purely endoscopic	GTR		6	No	/
50	PCP	M, 31	S/SS	2.5	Round	Cystic	No	/	Tight	Open	GTR		12	No	/
51	PCP	F, 31	Infundibulo-tuberal	3.8	Elliptical	Cystic	No	/	Tight	Open	GTR		12	No	/
52	PCP	F, 10	Strictly 3V	4.0	Multilobulated	Cystic	No	/	Loose	Open	GTR		30	No	/
53	PCP	F, 51	S/SS-secondary 3V	3.0	Round	Cystic	No	/	Tight	Open	GTR		24	No	/
54	PCP	M, 50	S/SS-secondary 3V	2.5	Elliptical	Cystic	No	/	Tight	Open	GTR		26	No	/
55	PCP	M, 57	S/SS-pseudo 3V	4.0	Pear-like	Cystic	No	/	Tight	Open	GTR		18	No	/
56	PCP	M, 48	S/SS-pseudo 3V	2.9	Round	Cystic	No	/	Tight	Open	GTR		30	No	/
57	PCP	M, 68	Strictly 3V	3.0	Round	Solid	Present	Big, popcorn	Invasion	Open	GTR		24	No	/
58	PCP	M, 28	S/SS-secondary 3V	7.0	Elliptical	Cystic	No	/	Invasion	Open	STR/PTR		36	Yes	24
59	PCP	M, 25	Infundibulo-tuberal	5.3	Round	Cystic	No	/	Invasion	Purely endoscopic	STR/PTR		24	Yes	6
60	PCP	M, 50	Strictly 3V	3.7	Elliptical	Cystic	No	/	Tight	Open	STR/PTR		12	Yes	2
61	PCP	M, 64	S/SS-pseudo 3V	4.8	Round	Cystic	No	/	Fusion	Purely endoscopic	NTR	pre OMMAY CI	15	Yes	9

S/SS: sellar-suprasellar; 3V: the third ventricle; Mixed: mixed solid-cystic; GTR: gross total resection, NTR: near-total resection, STR/PTR: subtotal resection/partial resection; RT: radiotherapy; pre OMMAY CI: Pre-OMMAY Capsule Implantation.

Table S2

Table S2. The correlation between ki-67 L.I. with ACP and PCP recurrence.

Histologic type	N=16	Ki-67 L.I.		X²	P
		0-4	≥5		
	No.（%）	No.（%）	No.（%）		
ACP	11(69)	3(27)	8(73)	0.097	0.453
PCP	5(31)	1(20)	4(80)		

L.I.: labeling index.

Table S3

Table S3. The relationship between Ki-67 expression and recurrence with the degree to CP adhesion and tumor resection.

Variables	N=59	Adhesion strength		X²	P value	Extent of tumor resection		X²	P value
		Loose+Tight	Invasion+Fusion			GTR	Non GTR		
	No.（%）	No.（%）	No.（%）			No.（%）	No.（%）		
Ki-67 L.I.				3.276	0.103			5.217	**0.027**
0-4	25(42)	19(76)	6(24)			21(84)	4(16)		
≥5	34(58)	18(53)	16(47)			19(56)	15(44)		
Recurrence									
Yes	16(27)	3(8)	13(59)	18.14	**<0.001**	2(5)	14(74)	30.746	**<0.001**
No	43(73)	34(92)	9(41)			38(95)	5(26)		

GTR: gross total resection; L.I.:labeling index.
